# What Has Finite Element Analysis Taught Us about Diabetic Foot Disease and Its Management? A Systematic Review

**DOI:** 10.1371/journal.pone.0109994

**Published:** 2014-10-07

**Authors:** Scott Telfer, Ahmet Erdemir, James Woodburn, Peter R. Cavanagh

**Affiliations:** 1 Institute for Applied Health Research, Glasgow Caledonian University, Glasgow, United Kingdom; 2 Department of Orthopaedics and Sports Medicine, University of Washington, Seattle, Washington, United States of America; 3 Computational Biomodeling (CoBi) Core, Department of Biomedical Engineering, Cleveland Clinic, Cleveland, Ohio, United States of America; Tel Aviv University, Israel

## Abstract

**Background:**

Over the past two decades finite element (FE) analysis has become a popular tool for researchers seeking to simulate the biomechanics of the healthy and diabetic foot. The primary aims of these simulations have been to improve our understanding of the foot’s complicated mechanical loading in health and disease and to inform interventions designed to prevent plantar ulceration, a major complication of diabetes. This article provides a systematic review and summary of the findings from FE analysis-based computational simulations of the diabetic foot.

**Methods:**

A systematic literature search was carried out and 31 relevant articles were identified covering three primary themes: methodological aspects relevant to modelling the diabetic foot; investigations of the pathomechanics of the diabetic foot; and simulation-based design of interventions to reduce ulceration risk.

**Results:**

Methodological studies illustrated appropriate use of FE analysis for simulation of foot mechanics, incorporating nonlinear tissue mechanics, contact and rigid body movements. FE studies of pathomechanics have provided estimates of internal soft tissue stresses, and suggest that such stresses may often be considerably larger than those measured at the plantar surface and are proportionally greater in the diabetic foot compared to controls. FE analysis allowed evaluation of insole performance and development of new insole designs, footwear and corrective surgery to effectively provide intervention strategies. The technique also presents the opportunity to simulate the effect of changes associated with the diabetic foot on non-mechanical factors such as blood supply to local tissues.

**Discussion:**

While significant advancement in diabetic foot research has been made possible by the use of FE analysis, translational utility of this powerful tool for routine clinical care at the patient level requires adoption of cost-effective (both in terms of labour and computation) and reliable approaches with clear clinical validity for decision making.

## Introduction

Ulceration occurring at the plantar surface of the foot in people with diabetes remains common, debilitating, difficult to treat, and costly [1]–[3]. In the absence of ischemia, neuropathy and the subsequent loss of protective sensation have been shown to be the main risk factors for the development of a first ulcer [4]. This, combined with repeated stresses on areas around bony prominences where protective tissues have been displaced [5] or have had their mechanical properties altered by the disease [6], is thought to be a key mechanism for ulcer development [7].

The complexity of the foot – both in terms of anatomy and function – and the difficulty in performing *in vivo* measurements over the full range of physiological stresses and strains that occur during functional loading have led researchers to seek ways of replicating its behaviour via computer-based simulations. For the diabetic foot, perhaps the most popular of these techniques is finite element (FE) analysis (also known as the finite element method), a form of simulation that allows the numerical solution of differential equations to predict the deformation field [8]. In practical terms, and as it relates to foot biomechanics, FE analysis allows us to find answers to complicated physical problems such as determining the response of the foot and the individual tissues that make up its anatomy to applied non-uniform loading.

FE analysis has proven to be a powerful tool for a number of engineering applications, and has been utilised by researchers carrying out a wide range of biomechanical investigations [9], [10]. An FE model produced for a biomechanical investigation consists of virtual representations of individual anatomical structures including, for example, bones and various soft tissues. These structures are each sub-divided into a large number of discrete but connected elements. While the overall geometry of each part may be complex, the geometry of the individual elements which together approximate its shape is not. Because of this, if the material properties of the element represents are known, the effects of applying a load, be it mechanical, thermal or other, to the individual element can be easily determined. By translating these effects to the surrounding elements, the effects on the larger more complex model can be calculated. Through this approach, numerous variables can be studied, such as contact pressures, internal stresses and strains, and energy transfers. In addition, it is possible to include non-biological components - for example an implant, an orthoses, or footwear - in the model, and thus simulate their effects and performance.

A fundamental advantage of the FE analysis approach for foot and footwear mechanics is its capacity to conduct parametric studies, which can help provide insights into foot function as a result of tissue behaviour, or can allow virtual prototyping, i.e. by varying the material properties or geometry of an insole. For researchers studying the biomechanics of the diabetic foot, the ability to generate these types of highly controlled results without the need for difficult and costly *in vivo* or cadaveric experiments makes FE analysis highly appealing.

This review of the literature is intended to provide a systematic overview of research that has used FE analysis to improve our understanding of the pathomechanics of the diabetic foot and to inform its management. Methodological considerations of the approach are addressed and insights gained are summarised.

## Methods

### 1. Search strategy

PUBMED and Web of Science databases were searched for relevant peer reviewed articles using the keywords “diabetic”, “foot”, “finite element” and related synonyms on November 25th, 2013. A representative search strategy is provided in Appendix S1. There were no limitations on publication dates but only English language articles were eligible for inclusion. Reference lists were examined for additional, relevant articles.

### 2. Study selection

This review considered original research studies that utilised FE models of the foot or part of the foot to simulate function, tissue behaviour, or structural deformities associated with the diabetic foot disease. In addition, studies using FE analysis to study footwear, insoles, or surgical interventions intended to reduce ulceration risk in people with diabetes were eligible for inclusion. Abstracts were screened and relevant articles selected for full text review. Full text papers were then reviewed to determine if they met the inclusion criteria. Screening and reviewing of articles was carried out by a single researcher (ST). For cases where it was unclear if the article met the inclusion criteria, a second researcher (AE) assessed the full text and the decision on whether or not to include the article was resolved by discussion between the review authors.

## Results

**Figure 1:**
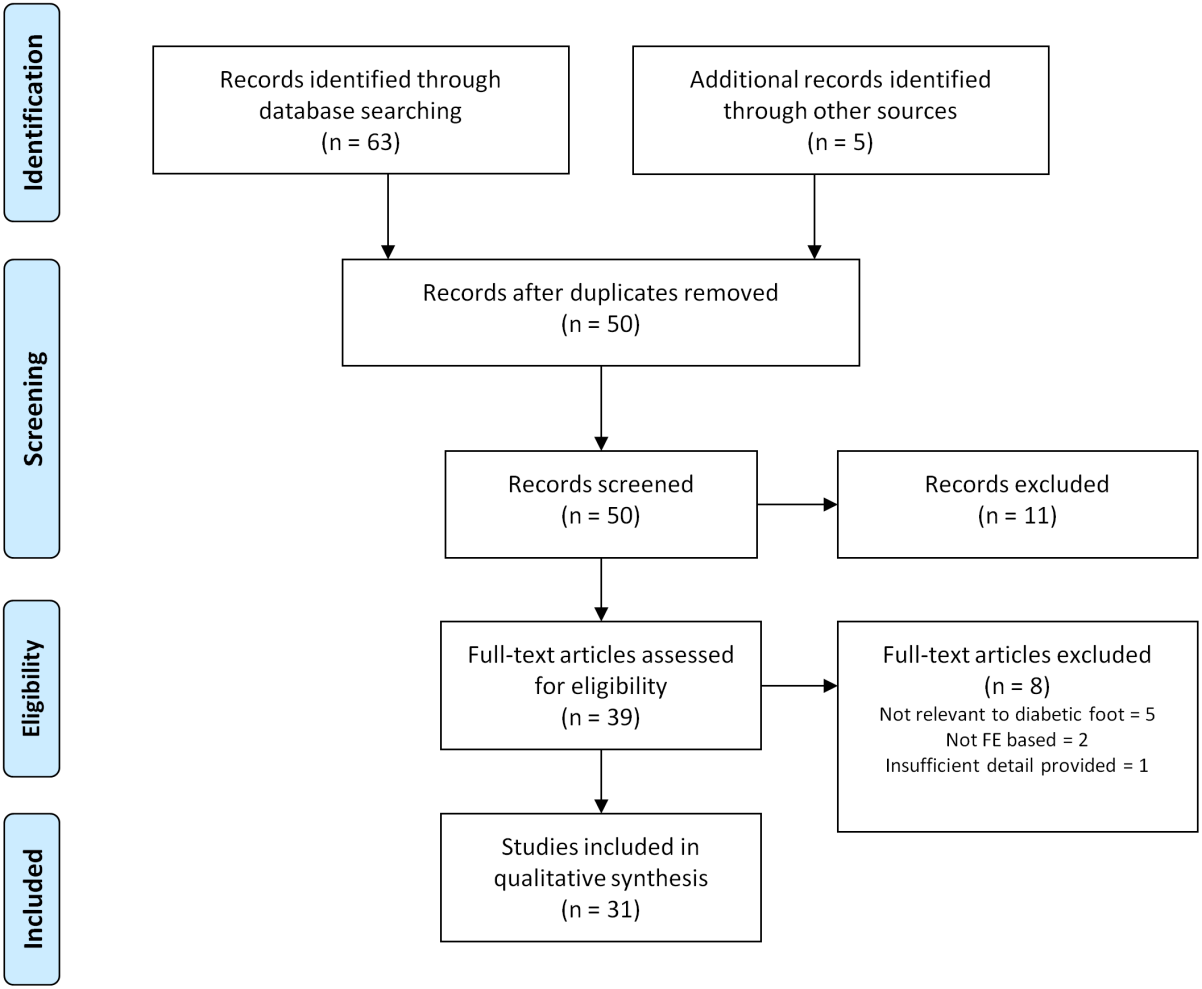
Flow of studies. From the initial 68 citations identified, 37 were excluded after removal of duplicates, initial screening of abstracts and review of full texts.

Fifty unique articles were identified from the literature search and of these, 31 met the eligibility criteria for inclusion (Figure 1). Articles were divided into three thematic groups: 1) studies on the methodological aspects of FE analysis of the diabetic foot; 2) investigations of the pathomechanics of the diabetic foot and; 3) the design of interventions intended to reduce ulceration risk. The included articles are summarised by group in Tables 1–3 respectively. In addition to discussing methodological aspects of modelling the diabetic foot, this article also presents a brief overview of the typical processes used to develop a model of the diabetic foot (or part of the foot) based on the reviewed literature.

**Table 1:** Studies on the methodological aspects of modeling the diabetic foot.

Article	Model			Validation	Key finding(s)
	2D/3D	Anatomy/Components(ground/floor alsomodelled unlessotherwise stated)	Simulation		
Petreet al., 2013	3D	Metatarsals, phalanges,muscle (NL), fat pad,(NL), skin (NL)	Inverse models todetermine materialproperties offorefoot softtissue. ModelA: bulkencapsulatedsoft tissue;model B: layersof soft tissue	Percentage errorin fit of optimisedmaterial propertiescompared to MRIand plantar pressuremeasurements = 5.1%.MSA = Y.	The internal stressesand deformationspredicted by the modelare affected by theinclusion of multiplelayers of soft tissue.Small changes inmaterial coefficientscan have large effectson tissue strain
Tadepalliet al., 2011	3D	Geometric representationof calcaneus, heel pad(NL) and insole.	Compressiveand combinedcompressive/shearloading	No validationexperiment includedin protocol.MSA = Y	Run times and resultvariations for differentelement types andloading conditionsproduced
Actis etal., 2006	2D	2 cross-sectional planesthrough the 2^nd^ and 3^rd^ raysof the foot includingrelevant foot bones,cartilage, encapsulated softtissue (NL) flexor tendonand plantar fascia, shoewith total contact insole	Six models ofdifferent complexityrun to assesseffect onpredictionat push-off	Correlation (r), bias(b) and dispersion(SD) of pressureprofiles on areasaround 2^nd^ and 3^rd^met heads.Meanr = 0.885;minr = 0.7;meanb = 16.6;maxb = 38.1;meanSD = 56.6;maxSD = 150.9.MSA = Y	For accurate simulationsof push off (barefootand shod) FE modelsrequire rearfoot,forefoot and toesegments separated bycartilage, flexortendon, plantar fasciaand soft tissue withNL properties.
Yarnitzkyet al., 2006	2D	Heel and 1^st^ metatarsalplantar soft tissue (L) usingFE analysis and combinedwith higher order analyticalmodel of rearfoot, forefootand hallux.	Real timeanalysis duringgait.	Compared withresults frompublished dataand phantomfoot with siliconrepresenting softtissue. Meandiff = 10%; maxdiff = 17%. MSA = NR	Proof of conceptprototype ofreal-time FE analysissystem

L: linear; NL: non-linear; MSA: mesh sensitivity analysis; NR: not reported.

**Table 2:** Investigations of the pathomechanics of the diabetic foot.

Paper	Model			Validation	Key finding(s)
	2D/3D	Anatomy/Components(ground/floor alsomodelled unlessotherwise stated)	Simulation		
Mithraratne Ket al., 2012	3D	Foot bones,muscles, softtissues (NL),arteries	Baseline and 3levels of tissuestiffening. Heelstrike, mid-stanceand toe-offmodelled.	As reportedin Fernandezet al. (2012)	Increasing softtissue stiffnessby a factor of 2reduces bloodflow to theaffected regionby 28%
Fernandez et al.,2012	3D	Foot bones,muscles, softtissues (NL),nerves	Baseline and 3levels of tissuestiffening. Heelstrike, mid-stanceand toe-offmodelled.	Peak plantarpressures (PP) andcontact areas (CA).PP mean diff = 7.7%;max diff = 10.1%; CAmean diff = 8.8%; CAmax diff = 14.3%.MSA = Y	Internal tissuestress up to 1.6times greater thanplantar surfacestresses. Increasingtissue stiffness bya factor of 2.5increased plantarpressures by30–40%
Chenet al., 2010	3D	Bone, cartilage,ligaments(inc. plantar fascia),encapsulated softtissue (NL).Selected muscleforces applied	Balancedstanding	Peak plantarpressures. Meandiff (based on 3sites) = 14.1%; Maxdiff = 29.2%(more sites tested butnot reported).MSA = Y	Internal stressescan be up to3 times greaterthan thosemeasured on theplantar surface
Gu et al.,2010	3D	Rearfoot bones,fat pad (NL)and skin (NL)	a) Inverse FE modelused to determineskin propertiesb) 4 levels of skinstiffness modelledduring heel strike	Peak pressureduring heel strike.MeanDiff = 6.3%.MSA = Y	Increasing skinstiffness by afactor of 3 leadsto an increase inpeak heel plantarpressure of 14.2%
Agicet al., 2008	2D	Cross-sectionalplane through the1st ray of the footincluding relevantfoot bones, cartilage,ligaments (NL),skin (NL) andencapsulated softtissue (NL)	Balancedstanding	No validationexperimentincluded in protocol.MSR = NR	Increased softtissue stiffnessleads to increasedpressure under theforefoot andrearfoot
Erdemiret al., 2006	2D	(a) 2D simplegeometry of heelpad and indentor;and (b) 2D coronal planeslice through calcaneusand heel pad (NL)	(a) Inverse model todetermine materialproperties viaultrasound indenter.(b) Tissue stiffnessand thickness variedwhile standing,walking and runningloads are applied toheel	a) Predicted vsmeasured force onindenter. Mean RMSerror for peak = 2.3%.b) Peak pressure andcontact area underheel: mean diff: 3.1%;mean diff 13.9%.Max diff = NR.MSA = NR	Tissue propertiesof diabetic heelsimilar to age andBMI matchedcontrols. InverseFE model todetermine materialpropertiesvalidated. Variation inindividual tissueproperties is such thatsubstantial errors whenusing generic properties
Cheunget al., 2005a	3D	All bones(phalanges fused)cartilage, all majorligaments andencapsulated softtissue (NL)	Stiffness of softtissue varied byfactors of 2, 3 and 5during balancedstanding	Peak pressuresbeneath heel and allmetatarsal headsduring barefootstanding. Meandiff = 42.1%; maxdiff 68.8%.MSA = Notreported.	Increasing tissuestiffness by 500%results in increasedplantar pressures of 35%at forefoot and up to80% increases in shearstresses Changes inbone stresses alsoreported
Thomaset al., 2004	3D	Foot bones(fused in medio-lateralplane into two arches),cartilage, majorligaments andencapsulated plantarsoft tissue (L).Selected muscleforces applied	Different soft tissuestiffness and thicknesssimulating healthy anddiabetic conditionsduring pushoff phase.	No validationexperimentincluded inprotocol.MSA = NR	For simulations of the diabetic soft tissue, plantar normal and shear stresses increased by up to52.3% and 53.6% respectively. Stress gradient ratios also increased in the diabetic simulations
Gefen2003	2D	5 cross-sectionalslices modelledcorresponding tofoot rays. Includesfoot bones, cartilagemajor relevantligaments, heel(NL) and metatarsalpads (NL)	Increasing stiffnessof soft tissues tosimulate changesassociated withdiabetes (5 levels)during balancedstanding.	No validationexperimentincluded inprotocol.MSA = Y	With tissue stiffening,contact forces at1^st^ and 2^nd^ met headincreased by up to 50%;internal tissue stressesincreased by up to 307%
Jacobet al., 1999	3D	Foot bones(fused in medio-lateralplane into two arches),cartilage, majorligaments andencapsulated plantarsoft tissue (L).Selected muscleforces applied	Healthy and diabeticfoot models tested(diabetic foot withdifferent soft tissueproperties). Heelstrike, mid-stanceand push off	Peak plantarpressures at 9locations for bothconditions. Meandiff = 11.9%; maxdiff = 37.1%.MSA = NR	Elevated plantar stressesfound in for the diabeticcondition compared tothe control

L: linear; NL: non-linear; MSA: mesh sensitivity analysis; NR: not reported.

**Table 3:** Investigations of interventions intended to reduce ulceration risk.

Article	Model			Validation	Key finding(s)
	2D/3D	Anatomy/Components(ground/floor alsomodelled unlessotherwise stated)	Simulation		
Isvilanondaet al., 2012	3D	Foot bones,cartilage, fat volumes(NL) and encapsulatedsoft tissue (skin and muscle,NL), tendons and ligaments.Selected muscle forces applied	Model modifiedto produce clawedhallux deformity. 2corrective surgicaltechniques thensimulated	Validated toranges reportedin literature.MSA = Y	Multiplescenarios maylead to clawedhalluxdeformity,with differentsurgicaltechniquesidentified asmoreappropriate incertain scenarios
Chen et al.,2012	3D	Bone, cartilage,ligaments(inc. plantar fascia),encapsulated softtissue (NL). Selectedmuscle forces applied	Gastroc-Soleusforce varied from100% (baseline) to60% in 10% stepsat instance offorefoot peakloading	Peak plantarpressures at metheads and toes.Mean diff = 13.6%;max diff = 38.5%.MSA = As reportedin Chenet al. (2010)	Pressuredistributionchanges at theforefoot causedby changingG–S force arenon-systematic.
Luo et al.,2011	2D	Calcaneus (rigid),skin (NL), heelpad (NL) andinsole designs	Barefoot, flatinsole, flat insolewith two designsof heel cutout,and customcountered insolesunder 25% bodyweight loading(approximatingbalanced standing).Insole stiffnessalso varied bythree levels	Mean plantarpressure at theheel reportedto be consistentbetween FE andexperiment but noabsolute valuesgiven. MSA = Y	Customcountered insoles providedgreatest reductions instress, strainand strainenergy density.Internalstresses nearthe calcaneuswere up to 10times greaterthan plantarsurface stresses
Gu et al.,2011	3D	Foot bones,cartilage, plantarfascia, encapsulatedsoft tissue (NL),midsole, insoleand heel plug	Thickness anddiameter of heelplug varied atinstance of heelstrike.	Peak plantarpressure at heel.Diff = 8.1%MSA = Y	Mediumhardness plug95% of the sizeof the calcaneuswas found toprovide mostpressure relief.
Shariatmadariet al., 2010	2D	Geometric simplificationof the calcaneus,encapsulated softtissue (NL), midsole(NL), and insolematerial (NL)	Barefoot and twoinsole materialconditions.Prescribeddisplacement tocalcaneus applied	No validationexperimentincluded inprotocol.MSR = NR	Temperaturecan have aneffect on insolematerialpropertieswhich in turnaffects theresulting footstresses
Shariatmadariet al., 2009	2D	Geometric simplificationof the calcaneus,encapsulated softtissue (L) and insolematerial	Barefoot and twoinsole materialconditions.Prescribeddisplacement tocalcaneus applied	No validationexperimentincluded inprotocol.MSR = NR	Peak stressoccurred at thecentral portionof the heel andgreater strainswere seen in theinsole material
Actis et al.,2008	2D	Cross-sectionalplane throughthe 2^nd^ ray of thefoot includingrelevant foot bones,cartilage, encapsulatedsoft tissue (NL)flexor tendon andplantar fascia, shoewith total contactinsole and smallinsole plugs	Variations oninsole modification(number of plugs,size of plugs,spatial distributionand plug material)at push-off phaseof gait	Correlation ofpressuredistributionaround metatarsalhead for bare foot(r = 0.83) and shod(r = 0.95).MSA = NR	FE used todetermine mosteffective plugdesign strategy.Inclusion ofplugs reducedpeak plantarpressure moresothan theconformingcustom insolealone
Cheung &Zhang 2008	3D	All bones(phalanges fused),cartilage, all majorligaments andencapsulated softtissue (NL), insole	Range of insolematerials andinsole designfactors tested atmidstance	No directvalidationexperimentincluded inprotocol,although generaltrends betweenFe and experimental results weresimilar. MSA = NR	Arch supportand elasticityof insolematerial foundto be designvariables withthe greatesteffect on plantarpressures
Budhabhattiet al., 2007	3D	1^st^ metatarsal,hallux(both phalanges),encapsulated softtissue (NL),and insole	5 different insolematerials tested atpush off totoe-off	Peak plantarpressure andtiming of peakplantar pressurefor coarse mesh(CM) and finemesh (FM).PPCMDiff = 6.7%;PPFMDiff = 58.3%;timingCMDiff = 7%;timingFMDiff = 5%.MSA = Y	Compared tobarefoot,reductions inpeak plantarpressure of 18–69%(1^st^ met head)and 43–68%(hallux) werefound usingdifferent insolematerials
Dai et al.,2006	3D	Bones combinedinto foot segmentsand divided bycartilage alongwith encapsulatedsoft tissue (L),sock and insole	Barefoot andtwo conditionswith frictioncoefficientsvaried to simulatedifferent sockmaterials. Footflat to push-off	Peak plantarpressures forefootand heel. Meandiff = 187.7%; maxdiff = 198.5%.MSA = NR	Reductions inplantar shearforces of >80%were foundwhen wearingsocks incomparison tothe barefootcondition
Goske et al.,2006	2D	Coronal planecross section ofheel includingcalcaneus,encapsulatedsoft tissue (NL)and insole	27 variations oninsole design: levelof conformity,thickness andmaterial. Averageheel loadingduring stanceused for simulation.	Peak pressureunder heel. Meandiff = 26.6%; maxdiff = 39.8%.MSA = Y	Level of insoleconformitymost importantvariable forreducing plantarsurfacepressures.
Erdemir et al., 2005	2D	Sagittal cross-sectionof 2^nd^ metatarsal,encapsulatedsoft tissue (NL)and midsole.	36 variations onpressure relief plugdesign: geometry,material andlocation simulatedat instance ofmaximumforefoot load	Peak pressureunder 2^nd^ met incontrol condition.Mean diff = 23%;max diff = NR.MSA = Y.	Locating plugsusing plantarpressuremeasurementsresults ingreater plantarsurface pressurereductions.Tapering ofplugs reducesedge effects
Cheung &Zhang., 2005	3D	Foot bones(phalanges fused)cartilage, all majorligaments andencapsulated softtissue (L). Flat andcustom insoles.	Flat and custominsoles withdifferent materialproperties duringbalanced standing	Peak plantarpressures atheel and forefoot.Mean diff = 100.4%;max diff = 131.4%.MSA = NR	Reductions inpeak plantarpressures of40.7% and31.6% found atforefoot andheel respectivelywhen using softcustom insolecompared to flatrigid.
Barani et al.,2005	3D	Insole (NL)	Four insolematerials testedwith loadingapplied at pointscorresponding tohallux, four siteson forefootand heel	No validationexperimentincluded inprotocol.MSA = NR	Silicone gelmaterialprovidedmaximumreductions instressconcentrationsand improveduniformity ofstressdistributions
Lewis 2003	2D	Rocker shoe design	Two variationson materialconfiguration foroutsole tested.Vertical pointloads approximatingstatic standingapplied	No validationexperimentincluded inprotocol.MSA = NR	Differentmaterialconfigurationscaused localvariations at thefoot soleinterfacealthough meanand peak resultswere similar.
Chen et al.,2003	3D	Foot bones modelledas lateral andmedial column,phalanges merged.Encapsulated softtissue (L) andmajor ligamentsincluded. Flatand custom insoles.	Flat insole andtwo total contactinsoles of differentmaterial layercompositionsduring midstance	No validationexperimentincluded inprotocol.MSA = NR	Reduction inpeak and meannormal plantarsurface stressesup to 56.8%using totalcontact insolescompared to flatfor all regionsexcludingmidfoot andhallux
Lemmonet al., 1997	2D	Sagittal crossectionof 2^nd^ metatarsal,encapsulated softtissue (NL), insole(NL) and midsole (NL).	Differentthickness ofinsole and plantartissues simulatedat instance ofmaximum forefootload	Peak plantarpressure beneath2^nd^ met head.Mean diff (12conditions) = 6.4%,max = 13.2%.MSR = Y	Insoles reducedplantar pressureby maximum of29% and weremost effect forreduced tissuethickness

L: linear; NL: non-linear; MSA: mesh sensitivity analysis; NR: not reported.

### 1. Methodological aspects of FE investigations of the diabetic foot

FE analysis is potentially complex with a large number of decisions required within the process of developing a model. This section gives a brief overview this process and the examples of the choices made by researchers when producing simulations of the foot. This is intended as a generic workflow, similar to those used in other FE-based biomechanical studies, and it should be noted that the specific order and implementation of the events described may vary depending on the software used and other factors.

#### 1.1 Geometry definition

**Figure 2:**
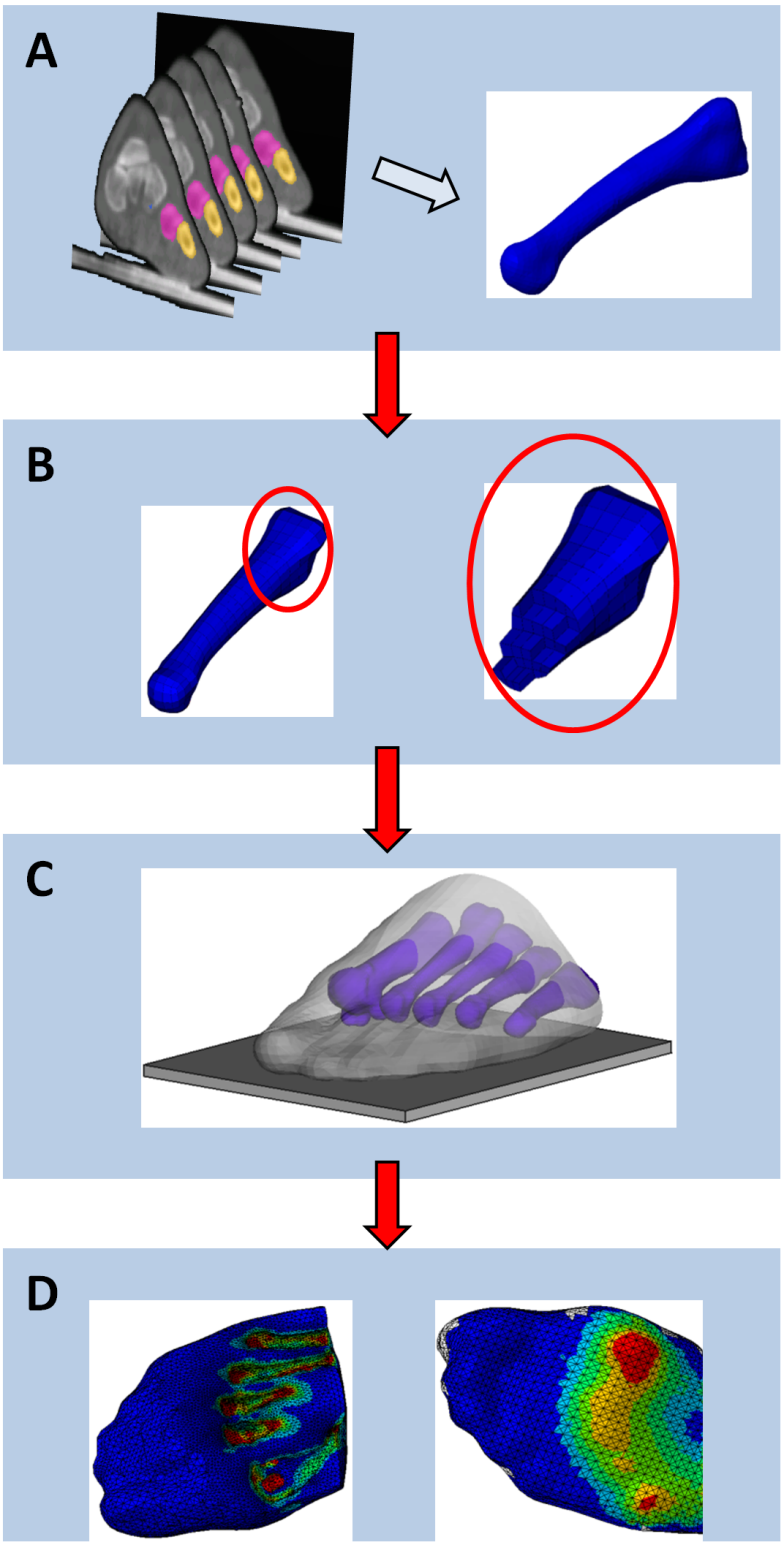
Development process for FE model of the foot. (A): Geometry of part constructed from CT slices of foot; (B): parts meshed into elements; (C): parts combined and interactions between them defined; (D): results of analysis, von Mises stresses (left) and plantar contact pressures (right).

The first stage of model development is to define the geometry of its constituent parts. Both 2D and 3D models have been included in this review, and these vary by their level of detail and personalisation. Two-dimensional models of the diabetic foot, generally representing a cross-section of the anatomy in the sagittal [11] or coronal plane [12], have the advantage of being faster to define and solve than 3D models, however, they cannot adequately represent any out of plane deformations of the tissue. For geometrically personalised 2D models, researchers have derived anatomical information on the structure of the foot from X-rays [13], computed tomography [14] and magnetic resonance imaging [15]. For accurate 3D representations of intrinsic anatomy, these have generally been reconstructed from the latter two imaging techniques by image segmentation as shown in Figure 2a [16], [17]. This can be a time and labour expensive step, although it may be automated to varying degrees depending on the accuracy required. Surface scanning or digitising of dissected parts of anatomy from cadavers after dissection has also been utilized [18]. If muscle forces or ligaments are to be included in the model, insertion and “via points” need to be defined from the imaging data and/or from standard anatomical references [17].

Deciding which anatomical structures to include in the foot model is an important question that needs to be addressed during the development process. Several groups have taken the approach of producing highly detailed models which attempt to include a large number of components including individual bones, ligaments, muscles, etc [18]–[20]. While this approach provides a more realistic representation of the foot, and hypothetically should produce more accurate results, the complexity of the model makes it time intensive both in terms of development and solution times, which in our experience can take several days, limiting their clinical utility.

For this reason, simplified models that do not represent the full anatomy but are intended to provide useful estimates in shorter timeframes have also been of interest to researchers [21], [22]. Actis et al. [14] carried out a range of simulations on a 2D model of the sagittal plane of the foot through the 2^nd^ and 3^rd^ ray to assess the effects of removing anatomy such as the plantar fascia and flexor tendon from their model. Results showed that modelling the phalanges of the toes as a single bone caused negligible changes to the model results, but that the inclusion of bones, cartilage, plantar fascia, flexor tendon and encapsulated soft tissue was necessary to provide accurate contact pressures at push off. Petre et al. [23] used an inverse FE model of the forefoot to study the effect of including different layers of plantar tissue - skin, fat pad and muscle - on the results. They found that representing all plantar tissue as a combined part with uniform material properties was adequate for predicting contact pressures; however internal stresses between layers could not be accurately predicted in this manner.

A further option, which has been reported in several of the studies included in this review, is the use of geometrically simplified models [21], [24]–[26]. These models utilise basic geometric shapes to represent the foot anatomy, for example a simple semi-circle to represent the plantar surface of the calcaneus in contact with the fat pad in the coronal plane [24]. While it is unclear if such models are able to provide accurate absolute patient-specific estimates values for model variables, this approach has the advantage of being quick to construct and solve, and can provide general insights into biomechanical characteristics [21] and FE simulation methodology [26].

#### 1.2 Meshing

The foot model then requires its individual components to be meshed, i.e. divided into discrete elements joined by nodes to allow it to be analysed (Figure 2b). Depending on the element geometry used and the complexity of the part, this process may be automated or may require significant user input. A range of element geometry was used in the studies included in this review including triangular [27] or quadrilateral [28] elements for 2D models and for 3D shell parts where only the surface is meshed [26], and tetrahedral [29] or hexahedral [30] elements for solid 3D parts. Within these basic shape groups, a number of additional options relating to the behaviour of the element can be assigned depending on the situation being modelled, for example reduced integration which can reduce run times at the expense of accuracy, or hybrid elements which are intended to model incompressible materials such as plantar soft tissue. Tadepalli et al. [26] tested a variety of elements for their suitability to model soft tissue behaviour (nonlinearly elastic, nearly incompressible or fully incompressible) in conjunction with footwear components and found that hybrid linear tetrahedral elements performed poorly when shear forces were applied, potentially limiting their suitability for studies on the foot. Hybrid linear hexahedral and quadratic tetrahedral elements reduced this problem, the latter at the expense of increasing the overall run time of the model and requiring the incompressibility restraint to be relaxed.

The density of the mesh (i.e. the number of elements making up the part) is an important factor if the model is to produce valid results. Using a converged mesh where the density is such that potential artefacts in the simulation results are minimised should give more accurate results. However the trade-off can be an exponential increase in the computational memory required and the time taken to solve the model. The approach adopted in many of the studies in this review is to perform a convergence analysis, i.e. to find a suitable mesh density by refining the discretisation until changes in the output metrics fall below a pre-defined acceptable level, suggesting that the density is sufficient to simulate the problem [11], [31], [32].

#### 1.3 Material properties

The next stage is for material properties to be defined for the various components of the model which, for the studies in this review, these are primarily related to mechanical behaviour. Properties can be drawn from existing literature [18] and in some cases combined with personalised data from *in vitro* or *in vivo* tests [14]. Additionally, to reduce complexity and increase model speed, some components may be defined as mechanically rigid, meaning that in the simulation they do not deform under load, for example Gu et al. [33] chose to model the ground in this way.

The plantar soft tissues of the foot in particular have been shown to have non-linear viscoelastic behaviour that may be highly variable between individuals [6], [12], [23]. A number of researchers, particularly in earlier studies, chose to define the plantar soft tissues in their models as having linear behaviour [13], [21]. However, all articles published since 2008 have used a non-linear material definition, generally Ogden hyperelastic, for plantar soft tissue. This compares to 43% of studies published prior to 2008 that used a non-linear formulation (see Tables 1–3). Other researchers have added additional hydrostatic constraints on the plantar tissues to allow the effect of tissue stiffening on arterial flow to be assessed [34].

Rather than defining the individual layers of skin, muscle, fat pad etc., many researchers have chosen to collapse these together as lumped models and to define the bulk properties of this combined encapsulated plantar tissue [19]. As described previously, this approach can provide accurate estimates of contact pressures, however it does not allow the interfaces between or stresses on individual layers to be studied [23].

#### 1.4 Interactions, boundary conditions and loading

Once the geometry of the parts have been defined it is necessary to characterise how they interact with one another. Models with more than one component are assembled and the parts positioned relative to each other as required (Figure 2c). Contact surfaces or tied interfaces between different tissues, connector definitions at joints etc., may be used depending on the model. To allow realistic simulation of the foot interfacing with an insole for example, the contact behaviour between the surfaces need to be defined, usually in form of friction coefficients. Researchers have used friction coefficients between the plantar surface and ground ranging from 0.3 [26] to 0.6 [18]. Application of concentrated or distributed loads to the model, in combination with boundary conditions to constrain the movement of parts in certain directions, allow the simulation of simplified or lifelike scenarios to deform the foot. In previous studies researchers have chosen to apply loading directly to the dorsal surface of the model [31] or in the form of force vectors attached to insertion points on bones that are intended to mimic the forces and action of selected muscles related to the simulation, attempting to drive the model in a more realistic manner [29].

#### 1.5 Analysis

The complexity and computational demands of simulating the foot throughout stance phase has meant that the majority of studies attempting to simulate the diabetic foot have used a quasi-static modelling approach, where the simulation is solved for a particular instance of stance phase (often the time of peak forefoot loading [31]) or repeated the simulation at several instances throughout stance phase [20]. A further commonly reported approach is to simulate the foot during balanced standing [11]. In each case the foot has to be orientated close to the analysis position prior to loading being applied. Attempts have been made to produce a real-time model suitable for clinical monitoring of the diabetic foot [21] using a hierarchical model which combined an analytical 2D three segment model of the foot (essentially hindfoot, forefoot and hallux) with 2D sagittal FE models of the plantar pad under the heel and distal metatarsal head.

FE models can generate a large amount of detailed information, and interpreting these data such that simple, clinically relevant metrics can be provided is important. In the articles included in this review, data was often provided visually as colour contour maps (as in Figure 2d) and simple magnitude-based variables such as Von Mises stresses [11], surface pressures [35] or shear forces [36] within anatomical regions of interest. Primary output measures used by the reviewed studies are listed in Tables 1–3.

### 2. Investigating the pathomechanics of the diabetic foot

In the studies included in this review, the effect of diabetes on the foot was primarily simulated by increasing the stiffness or reducing the thickness of the plantar soft tissues (see Table 2). A number of experimental studies have found differences in these variables when comparing patients with diabetes to healthy controls [6], [37]. However, it is worth noting that Erdemir et al. [12] when using an inverse FE model to determine heel pad tissue properties, found no statistical difference in tissue behaviour between the groups but high variability in the results.

Stresses on the plantar surface of the foot remain highly relevant in assessments of the diabetic foot and ulceration risk. Several FE studies have demonstrated increases in peak plantar pressures associated with stiffening and thinning of plantar tissues in both 2D [11], [27] and 3D [13], [19], [20], [33], [38] models of varying complexity. In addition to investigating surface stresses, FE simulations suggest that the internal stresses on plantar tissues may be several times larger [16] and increase at a greater rate with tissue stiffening [11], [20]. These studies included those that modelled the plantar soft tissues both as 2D bulk tissue [11], [16] and as 3D individual layers [20]. The bulk tissue models tended to predict larger increases in peak internal stresses for similar levels of stiffening, possibly reflecting the difficulties in accurately estimating these variables using a combined model [23]. In terms of location, peak internal stresses were predicted near the metatarsophalangeal joints [11], near the bony prominences of each metatarsal head [16], and under the 3^rd^ and 4^th^ metatarsal heads midway between the bone and plantar surface [20]. It should be emphasised however that the single-subject nature of the models, along with the abovementioned issues in modelling the tissue layers, means that caution should be applied if attempting to extrapolate these findings to the general or patient population.

FE analysis also allows plantar shear stresses to be estimated. This is a variable that can be difficult to measure experimentally but which has been suggested as a mechanism for plantar ulceration [39], however there is currently no prospective evidence to support this. Both Thomas et al. [13] and Cheung et al. [19] have found that when comparing control simulations to simulations of the diabetic foot with increased soft tissue stiffness and reduced thickness of plantar soft tissue, shear stresses could increase by >50% when compared to a control simulation during push off and balanced standing.

FE models can also be used in an inverse manner to determine the material properties of an individual’s soft tissue using a combination of imaging and force data. Erdemir et al. [12] used an inverse FE technique using data from an ultrasound indenter instrumented to also measure force and found that the mechanical properties of the heel pad in patients with diabetes were not statistically different from healthy controls although they reported considerable variability in the data. Beyond studies focusing directly on the foot tissues, FE models can be used to study other aspects of the diabetic foot pathomechanics. Mithrarante et al. [34] represented the vascular system in their 3D FE model of the foot and looked at the effect of increasing tissue stiffness to levels producing physiologically feasible hydrostatic pressures on this system to simulate the effects of diabetes. They found that by doubling the tissue stiffness, a reduction in blood supply of 28% was seen in affected areas.

### 3. Interventions to reduce ulceration risk

The majority of studies included in this review which investigated interventions intended to reduce ulceration risk considered some form of insole or footwear (see Table 3). A wide range of clinically used materials have been tested in FE simulations of insoles [15], [17], [24], [30], [31], [35], [40]–[42] and footwear [43]. Generally the elasticity of the footwear material was found to be the most influential factor in terms of pressure relief.

FE analysis has also been utilised to explore the effects of environmental influences on the performance of the insole material. Shariatmadari et al. [25] used a 2D coronal plane model of the heel to assess the pressure relieving performance of commonly used insole materials from 10–40°C. By modelling the known effects of temperature on material elasticity properties, the study showed that the changes in material performance within these temperatures had a considerable effect on the ability of these commonly prescribed insole materials to reduce peak plantar pressures, with performance being the worst at 10°C.

Using both 2D [15], [28] and 3D [17], [35] models it has been predicted that custom contoured insoles provide the greatest reductions in plantar surface pressures when compared to flat insoles made from pressure relieving materials. The inclusion of an arch support to an insole design was also found to be beneficial in terms of redistributing plantar pressures away from at-risk areas such as below the metatarsal heads [17]. In addition, Dai et al. [36] developed a model and insole that included a layer of material representing a sock. Using properties derived from experimental measures of different sock materials they predicted that, compared to barefoot, the use of socks can result in a reduction in plantar shear stresses of >80%.

Several studies have been conducted into pressure relieving plug modifications for insoles or footwear. These have looked at modifications at the forefoot [22], [40] and heel [33]. Erdemir et al. [40] demonstrated that significant reductions in peak pressures under the metatarsal head could be achieved using this strategy. In addition, the simulations showed that locating the plug via the peak of the pressure distribution was more effective in terms of reducing peak pressures compared to locating it directly under the metatarsal head. Actis et al. [22] expanded upon this work by using a 2D model of the full foot (cross section of the second ray) to test the effects of smaller (∼4 mm) plugs inserted into an area of a total contact insole under the metatarsal head. They found that while the small plugs did reduce peak plantar pressures, the magnitude of the reduction both found numerically and experimentally was lower than those reported by Erdemir et al. [40].

Gu et al. [33] looked at similar interventions at the heel and found that a medium hardness plug ≥10 mm thick with a diameter 95% of that of the calcaneus was the most effective at reducing plantar pressures. All three studies looking at this type of modification identified the risk of edge effects, where pressure concentrations are seen at the interface between the cut-out or plug and the stiffer material of the insole, and suggested strategies for reducing these.

In addition to footwear based interventions, FE analysis has also been utilised to investigate surgical techniques used to treat at risk individuals at risk for plantar ulceration. Isvilanonda et al. [18] used their model to examine clawed hallux deformity. They found several configurations by which the deformity could occur and the results from simulating surgical intervention suggested that certain surgeries may be more effective for reducing plantar pressures depending on the muscular imbalance that led to the deformity. The use of tendo-achillies lengthening to reduce ulceration risk in diabetic patients with equinus has also been investigated by FE analysis [29]. The authors found that the effect of reducing gastrocnemius-soleus force on plantar pressures to be inconsistent and no systematic effect was determined.

## Discussion

By utilising FE analysis, researchers have been able to provide a number of insights into the diabetic foot and its interventions, which would not be possible through *in vivo* or *in vitro* experimentation. The finding that the internal stresses in the plantar tissues can be considerably greater in the diabetic foot is potentially highly relevant to ulcer development, since the internal formation of some deep ulcers has been recognised clinically [44]. The results presented by these FE models provide a feasible mechanism for the development of this type of ulcer, and also potentially a modifiable biomechanical treatment target. Efforts to incorporate different components into the models such as the vascular system, which may also provide a contributory mechanism for ulcer development in diabetes, demonstrate the versatility of the technique and suggest interesting avenues for future work.

It is important to note that FE analysis provides a predictive platform where novel insole designs, potentially individualised for a patient’s anatomy, can be virtually tested before prescription. Despite different approaches to modelling the foot and levels of complexity, studies looking at different types of insole interventions suggested that custom contoured devices were the most effective of the designs studied at providing pressure relief. This agrees with the preponderance of evidence from studies using in-shoe plantar pressure measurement systems and is in line with current recommendations that a custom, pressure relieving insole should be prescribed for at-risk patients [4]. A recent randomised controlled trial comparing standard custom contoured insoles to those which incorporated plantar pressure measurements as well as shape into their prescription process found the shape and pressure devices to be more effective at preventing ulcer recurrence [45]. This may be supported by results from FE simulations showing that the locating pressure relieving plugs using the location of peak pressures produced greater reductions than those located based on the anatomical location of the metatarsal head alone [40]. The findings by Shariatmadari et al. [25] that temperature can affect the pressure relieving performance of insole materials also raises questions about insole performance in different seasons/climates and whether this should be taken into account when prescribing the devices.

The complexity of foot anatomy and function necessitates that a wide range of assumptions and simplifications are made in model development, and different opinions exist as to how complex a foot model needs to be in order to be clinically useful. The findings from this review do suggest that 2D models, when built carefully in relevance to the question that needs to be addressed, may be able to provide useful results [22], [28], [31], however further work and evaluation in patient populations is required to understand their general utility. The results do indicate that, given the variability in tissue properties in both diabetic and healthy plantar tissue, an important part of producing clinically useful patient-specific FE simulations may involve accounting for these variations in material behaviour within the model. A range of approaches for collecting and analysing this type of data have been suggested [12], [46].

Simulating the diabetic foot by increasing the stiffness or reducing the thickness of plantar tissues may be another valid approach. However further biomechanical alterations in the diabetic foot, for example, increased joint stiffness [47] have yet to be incorporated into FE models and may be a further avenue for research.

Presently, the generation of an anatomically detailed 3D model of a foot from imaging data is a time and labour intensive process and is not feasible for individual patients. Approaches using template models that can be parametrically scaled to match the foot anatomy of different individuals based on simpler measurements have been suggested [48]. This may be a more feasible approach but it remains to be fully realised.

The development of an FE model of the foot requires considerable simplification of complex anatomy and mechanics. The elements used, the definition of interactions, and the representation of material behaviour are only some of the decisions that need to be made where there may not be a single “correct” answer with sound justifications. Some of the studies included in this review reported only a modest amount of information regarding limitations and validation making replication of the work difficult. Detailed reporting guidelines by Erdemir et al. [49] have recently been published to complement existing recommendations [50] and if these were to be adopted by researchers in the field, it would help to better standardise the information provided in publications. Where possible, making models openly available may also help to advance the approach.

To ensure confidence in the results, confirmation of simulation results against experimentally derived data should be a key part of any FE study. In the studies included in this review, the majority compared peak plantar pressures from the model to those collected experimentally at one or more sites, however in many cases no clear evaluation protocol was included in the modelling and simulation approach. In addition, comparisons between surface pressures from FE models to experimentally collected data can be confounded by differences in the measurement resolution of the methods, particularly in the case of in-shoe measurements where the resolution can be relatively poor in comparison to the model.

Several studies compared their results to those reported in the literature as validation. In the authors’ opinion, whenever possible this approach should only be used as a secondary validation measure. We recommend that the method chosen to validate the model should be defined *a priori* and, if possible, should not rely on a single variable. Reporting model comparisons with a number of different variables may provide greater confidence in the predictive value of the model for those variables it is not possible to measure directly. Ideally the primary researchers should be blinded to at least some of the validation results during the model development process.

In addition to the methodological issues identified above, there are a number of more general barriers to the increased use of FE analysis for investigations related to the diabetic foot. FE foot models, particularly those in 3D, can be demanding to build and run with simulation times that can last many hours or even days depending on the complexity of the model. To improve the clinical utility of FE analysis both model development and simulation times may need to be significantly reduced, and some model simplification or reduction procedures have been suggested which have yet to be evaluated for their clinical validity. Intensive training is required to reach a suitable level of expertise to be able to develop models and interpret their results. Finally, high-end solvers for FE analysis remain expensive, although in recent years a number of open source FE and supporting programs have been developed, potentially allowing greater access to the technology (for example, see FEBio, http://www.febio.org/ and Calculix, http://www.calculix.de/).

## Conclusion

The application of FE analysis to study the biomechanics of the diabetic foot has resulted in a number of insights regarding its pathomechanics and has aided the design of interventions. However, considerable progress will be required before the technique can be utilised outside of the research domain to inform clinical management of diabetic foot disease at the level of the individual patient.

## Supporting Information

Appendix S1 **Search strategy.**
(DOCX)

Checklist S1 **PRISMA checklist.**
(DOCX)
