# Supplementary material for: What Has Finite Element Analysis Taught Us about Diabetic Foot Disease and Its Management? A Systematic Review
Source: PLoS One. 2014 Oct 7;9(10):e109994. doi: 10.1371/journal.pone.0109994 (PMC4188702; doi:10.1371/journal.pone.0109994)
Supplement: Appendix S1 — Search strategy. (DOCX) [file pone.0109994.s001.docx]

**Appendix S1**

***Search strategy***

The literature search was carried out 1^st^ to the 5^th^ of November, 2013. The following strategy was used in PUBMED and syntax adapted for other databases-

("foot"[MeSH Terms] OR "foot"[All Fields]) AND (("diabetes mellitus"[MeSH Terms] OR ("diabetes"[All Fields] AND "mellitus"[All Fields]) OR "diabetes mellitus"[All Fields] OR "diabetes"[All Fields] OR "diabetes insipidus"[MeSH Terms] OR ("diabetes"[All Fields] AND "insipidus"[All Fields]) OR "diabetes insipidus"[All Fields]) OR diabetic[All Fields]) AND (finite[All Fields] AND ("elements"[MeSH Terms] OR "elements"[All Fields] OR "element"[All Fields]))
